# Supplementary material for: Single-point mutated lanmodulin as a high-performance MRI contrast agent for vascular and kidney imaging
Source: Nat Commun. 2024 Nov 13;15:9834. doi: 10.1038/s41467-024-54167-4 (PMC11561317; doi:10.1038/s41467-024-54167-4)

Supplementary Information for

Single-Point Mutated Lanmodulin as a High-Performance MRI Contrast Agent for Vascular and Kidney Imaging

Table of Contents **Page**

Fig. S1. Sequence of full-length lanmodulin protein. S3

Fig. S2. Comparative structural analysis of surface properties between LanM and LanND. S4

Fig. S3. SDS-PAGE analysis of LanND purification process. S5

Fig. S4. Assessment of metal-loading capacity via ICP-OES. S6

Fig. S5. Determination of the affinity of Fluo-5N to Gd^{3+}. S7

Fig. S6. Visual and SDS-PAGE comparison of proteins before and after Gd^{3+} ion loading. S8

Fig. S7. Characterization of the relaxivity performance of LanM-Gd at 3T. S9

Fig. S8. Evaluation of T_1 relaxation for LanND-Gd at 7T. S10

Fig. S9. Investigation of the T_2 relaxivities for protein-based contrast agents at 3T and 7T. S11

Fig. S10. Evaluation of potential Gd^{3+} leakage by Ca^{2+} imaging of Piezo1. S12

Fig. S11. Gd^{3+} binding affinity for LanND under various interference factors. S13

Fig. S12. Evaluation of cytotoxicity of LanND-Gd. S14

Fig. S13. T_1-weighted eye images enhanced by Magnevist or LanND-Gd under 3T. S15

Fig. S14. T_1-weighted brain images enhanced by Magnevist or LanND-Gd under 9.4T. S16

Fig. S15. Whole-body imaging contrasted with different contrast agents. S17

Fig. S16. T_1-weighted brain images enhanced by Magnevist or LanM-Gd under 9.4 T. S18

Fig. S17. Distribution of Gd^{3+} in various organs after injection of LanND-Gd. S19

Fig. S18. Characterization of GFP-tagged LanND. S20

Fig. S19. Emotional test for potential immunogenic damage. S21

Fig. S20. Colloidal stability of protein-based agents under environmental variations. S22

Supplementary Movie 1. 3D-reconstructed imaging before and after injecting LanND-Gd.

Supplementary Movie 2. 3D-reconstructed imaging for brain vasculatures.

Signal peptide

1 MAFRLSSAVLLAALVAAPAYAAPTT 25

EF-hand 1

26 TTKVDIAAFDPDKDGTIDLKEALAA 50

EF-hand 2

51 GSAAFDKLDPDKDGTLDAKELKGR 75

EF-hand 3

76 VSEADLKKLDPDNDGTLDKKEYLAA 100

EF-hand 4

101 VEAQFKAANPDNDGTIDARELASPA 125

126 GSALVNLIR

Supplementary Fig. 1 | Sequence of full-length lanmodulin protein. Lanmodulin features key components including a signaling peptide on its *N*-terminus, three well-defined lanthanide binding sites labeled as EF-hand 1, EF-hand 2, and EF-hand 3, alongside one lower ion binding affinity site denoted as EF-hand 4.

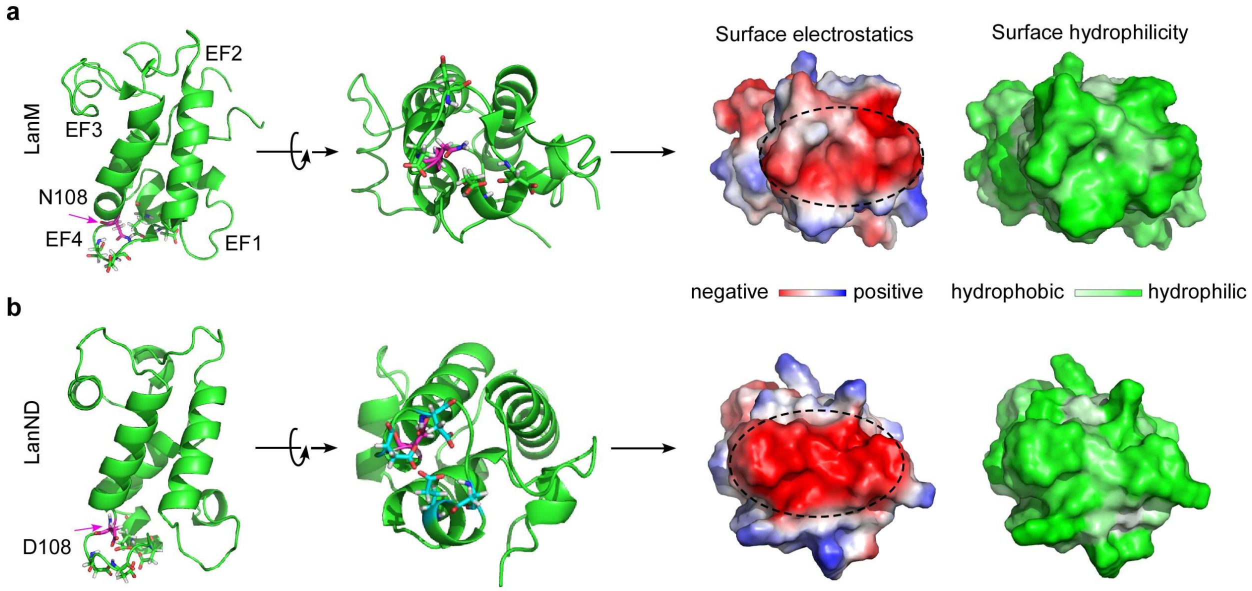

Supplementary Fig. 2 | Comparative structural analysis of surface properties between LanM and LanND. a, Structural properties of wild-type lanmodulin (LanM, PDB: 6MI5). **b,** Structural properties of lanmodulin_N108D (LanND), where the structure of LanND was predicted using AlphaFold2. All analyses were performed using PyMOL software.

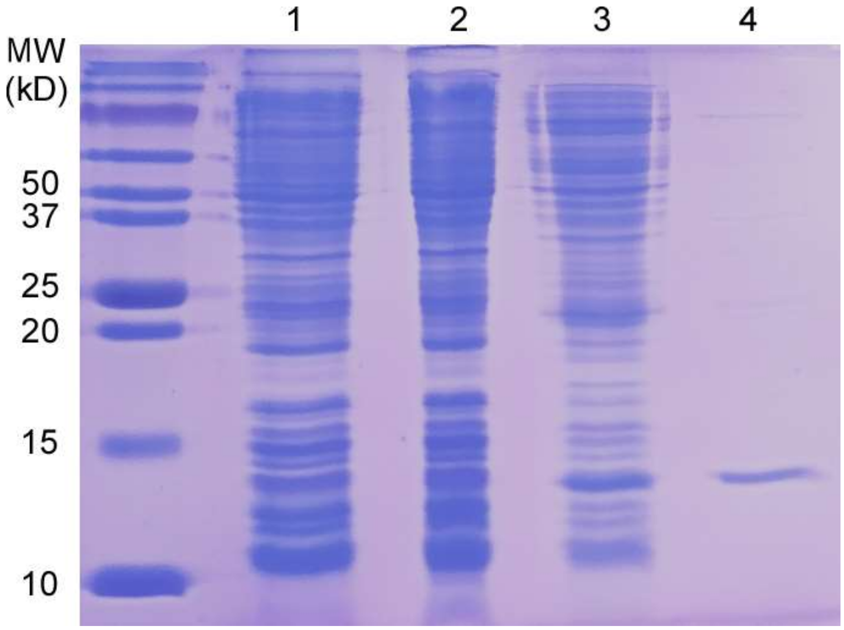

Supplementary Fig. 3 | SDS-PAGE analysis of LanND purification process. Lane 1: cell lysis sample. Lane 2: supernatant after centrifugation. Lane 3: wash-out solution. Lane 4: eluted proteins obtained during purification. Experiments were repeated three times, yielding similar results.

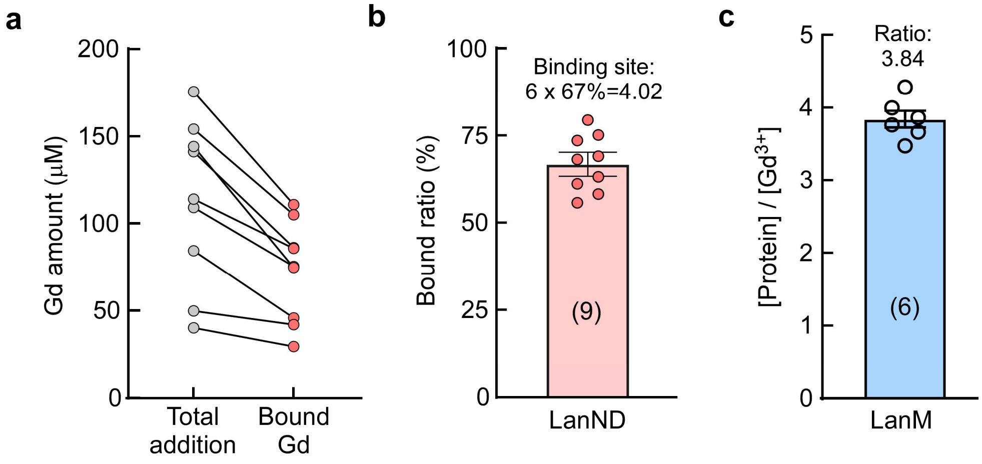

Supplementary Fig. 4 | Assessment of metal-loading capacity *via* ICP-OES. a, Gd amount in the systems before and after filtration. **b,** Statistical analysis of Gd ratios between post-treatment and prior-treatment of filtration. **c,** Statistical analysis of the ratio between LanM and Gd^{3+} after filtration. Gd^{3+} ions were loaded to LanM or LanND, followed by ICP-OES analysis to quantify Gd^{3+} concentration within the protein solutions. Note that buffer was exchanged using desalting columns and spin filters to remove any unbound Gd^{3+} ions. Data are presented as mean ± SEM in (b-c). Source data are provided as a Source Data file.

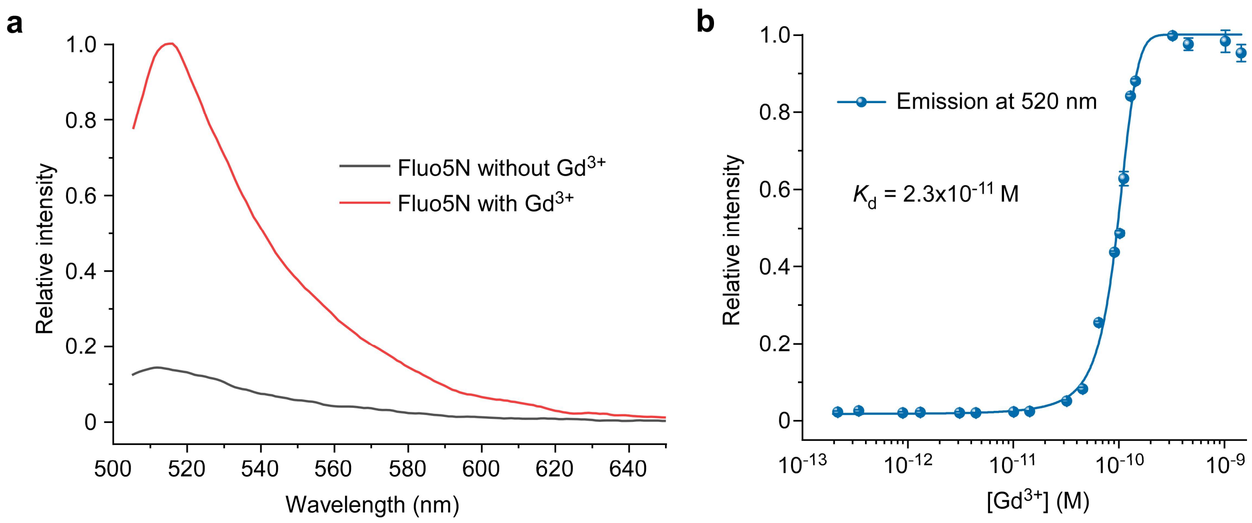

Supplementary Fig. 5 | Determination of the affinity of Fluo-5N to Gd^{3+}. a, Representative spectra of Fluo-5N (1 μM) in the absence or presence of $GdCl_3$ (1 μM). **b,** K_d determination for Fluo-5N by Gd^{3+} titration. With Hill fitting, the K_d was determined to be 2.3×10^{-11} M. Experiments were performed in biological triplicate and data are presented as mean ± SEM. Source data are provided as a Source Data file.

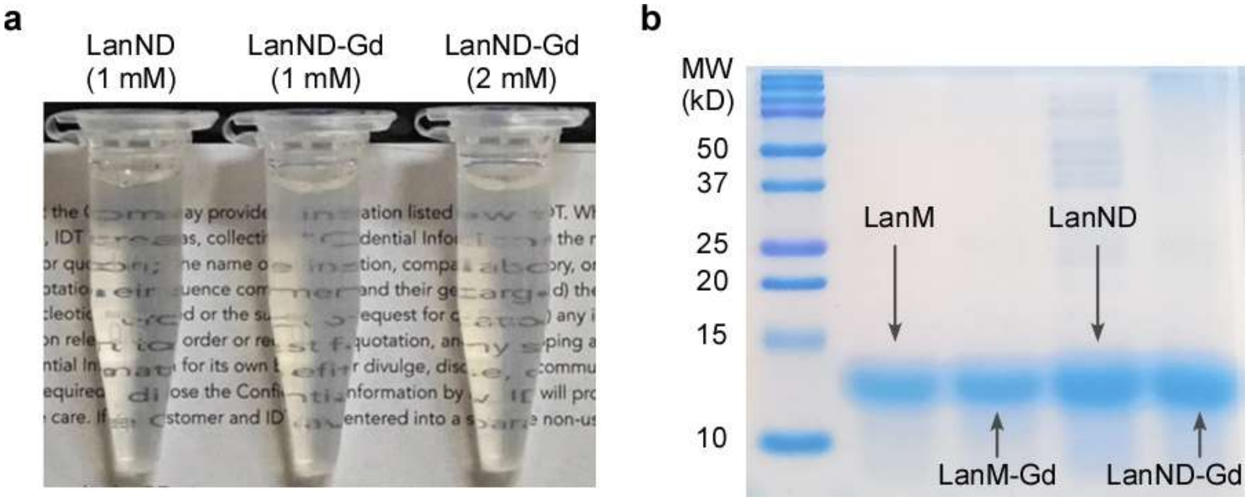

Supplementary Fig. 6 | Visual and SDS-PAGE comparison of proteins before and after Gd^{3+} ion loading. a, Comparative photographs of colloidal solutions containing apo-state proteins and Gd^{3+}-loaded proteins. **b,** SDS-PAGE analysis of proteins before and after Gd^{3+} ion loading. Experiments were repeated three times, yielding similar results.

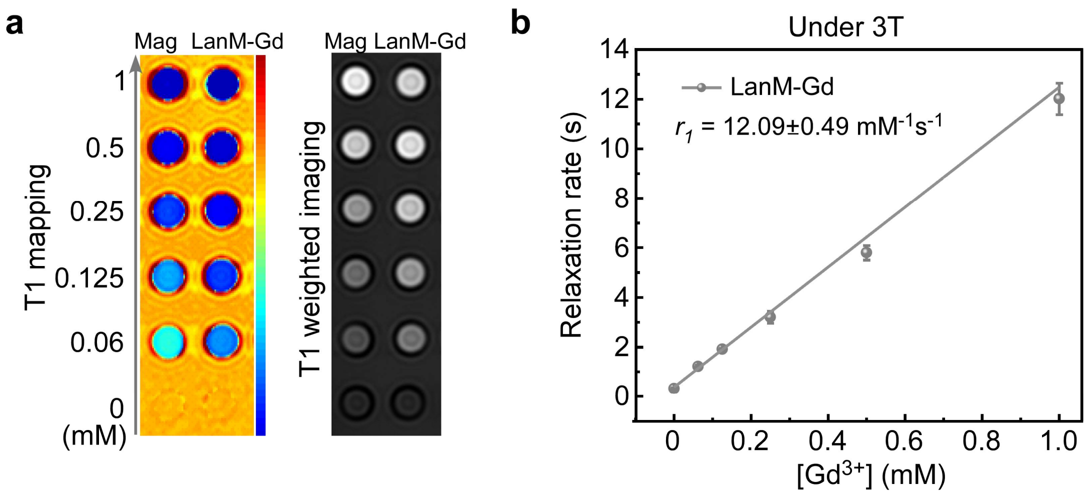

Supplementary Fig.7 | Characterization of the relaxivity performance of LanM-Gd. a, Comparison of T_1 mapping and T_1-weighted imaging between Magnevist (Mag) and LanM-Gd under a 3T scanner. **b,** Correlation between relaxation rates and Gd^{3+} concentrations. Data were obtained from three independent measurements, and relativities were determined by calculating slopes from linear fittings. Experiments were performed in biological triplicate and data are presented as mean ± SEM. Source data are provided as a Source Data file.

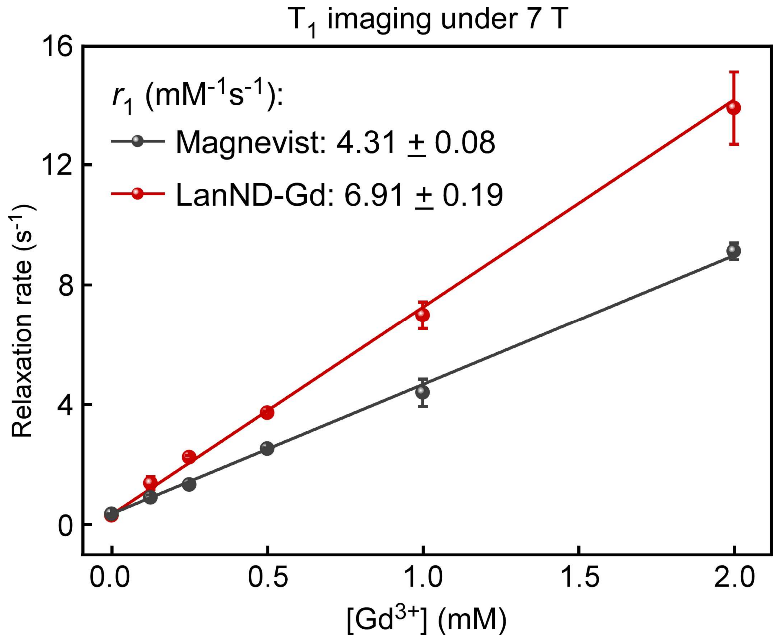

Supplementary Fig. 8 | Evaluation of T_1 relaxation for LanND-Gd at 7T. Correlation between R_1 relaxation rates and Gd^{3+} concentrations at 7T. Experiments were performed in biological triplicate and data are shown as mean ± SEM. Source data are provided as a Source Data file.

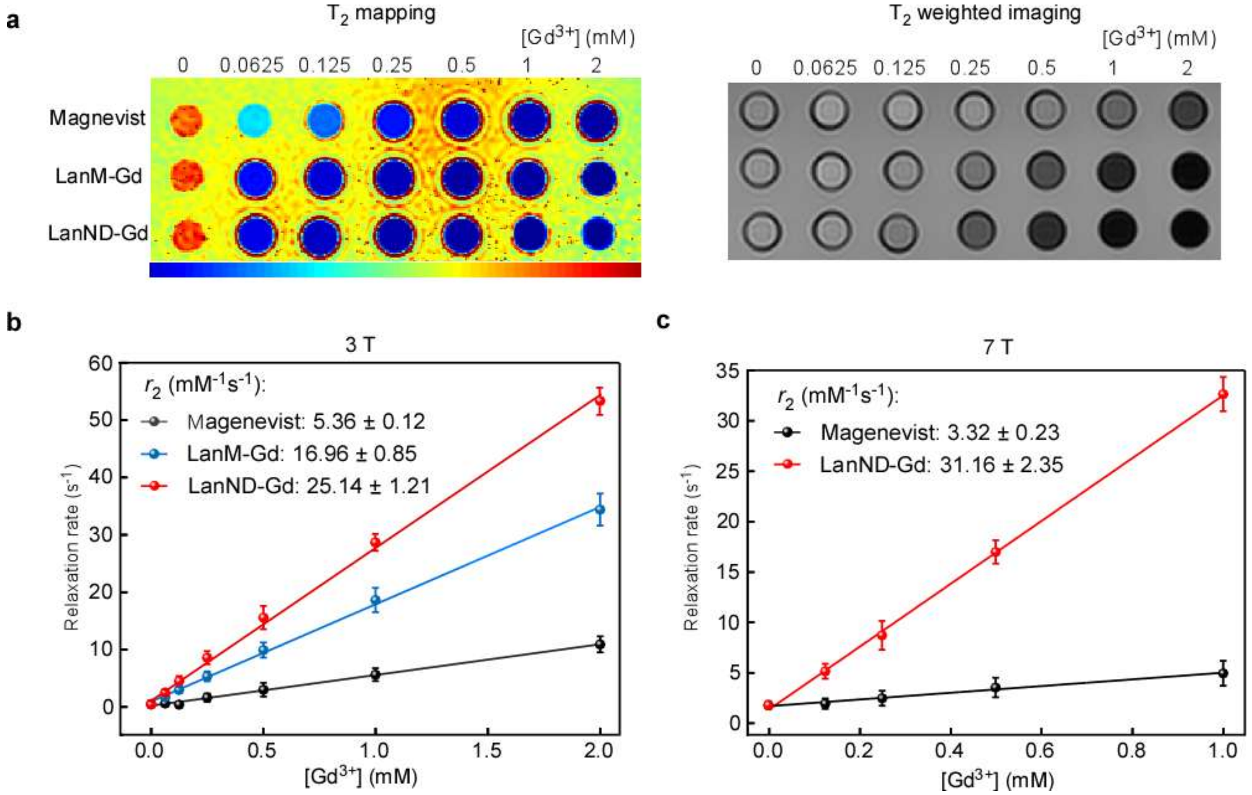

Supplementary Fig. 9 | Investigation of the T_2 relaxivities for protein-based contrast agents at 3T and 7T. a, T_2 mapping and T_2 weighted imaging comparing protein-based contrast agents and Magnevist. **b,** Changes in R_2 relaxation rates relative to Gd^{3+} concentration at 3T. **c,** Changes in R_2 relaxation rates with respect to Gd^{3+}concentration at 7T. Each point was independently measured three times. Data are presented as mean ± SEM in (b-c). Source data are provided as a Source Data file.

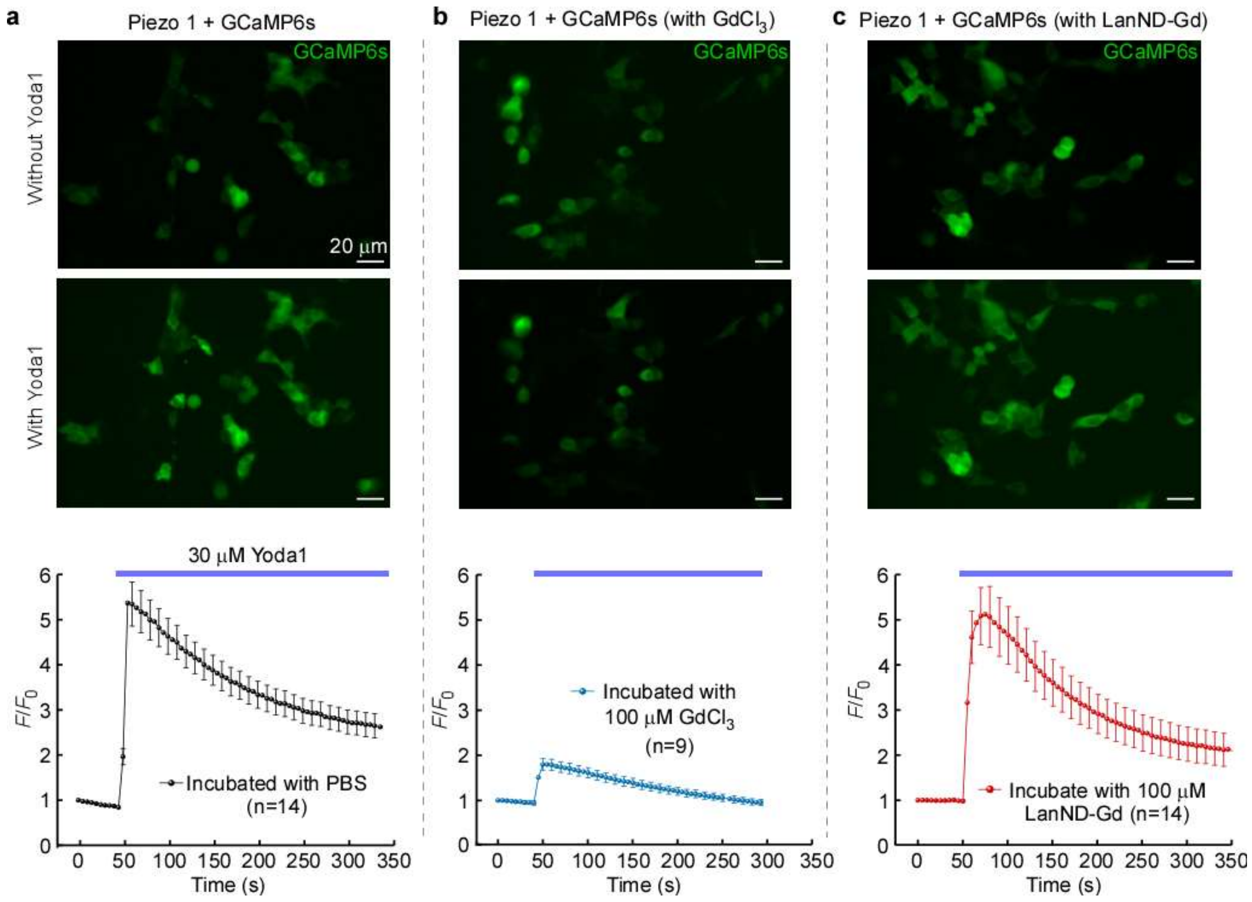

Supplementary Fig. 10 | Evaluation of potential Gd^{3+} leakage by Ca^{2+} imaging of Piezo1. The experiments involved incubation of Piezo1-expressing cells for 24 h under three different conditions: PBS (**a**), 100 μM $GdCl_3$ (**b**), and 100 μM LanND-Gd (**c**). Ca^{2+} influx *via* Piezo1 was induced by the channel agonist Yoda1 at a concentration of 30 μM using a rapid solution exchanger. Data are presented as mean ± SEM. Source data are provided as a Source Data file.

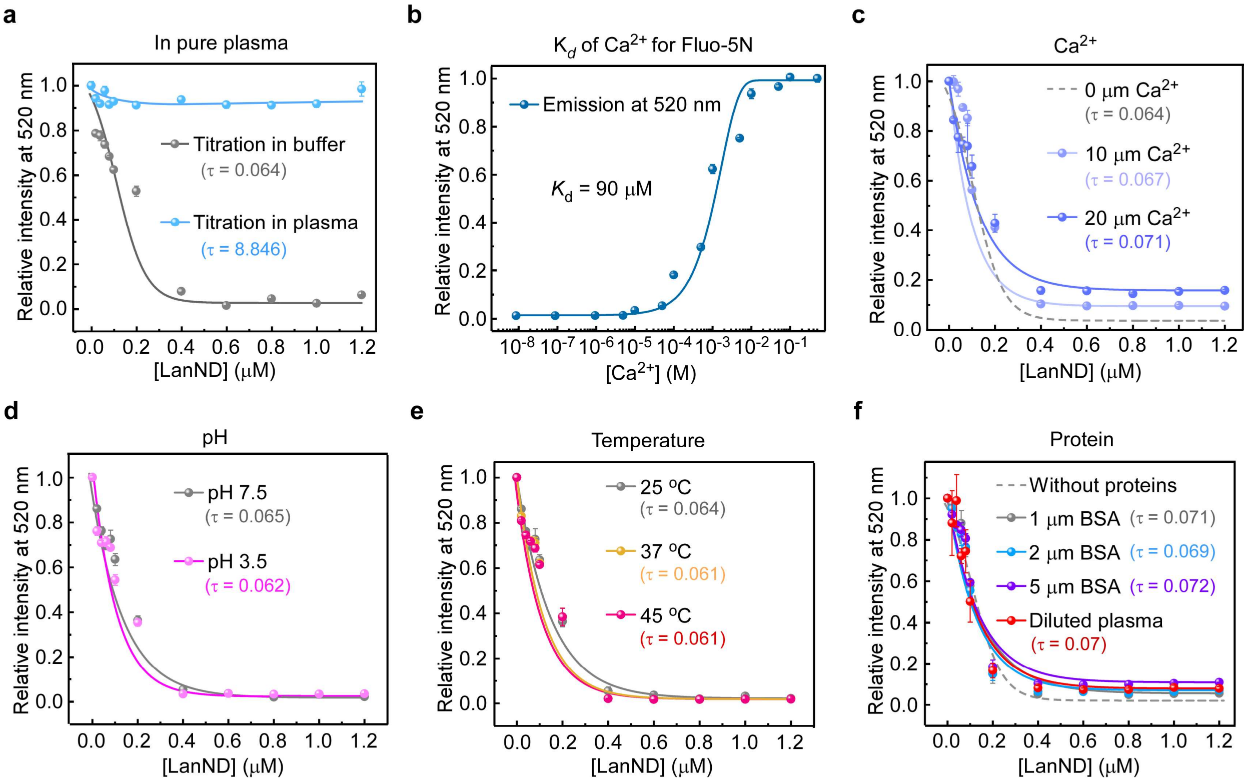

Supplementary Fig. 11 | Investigation of Gd^{3+} binding affinity for LanND under various interference factors. a, Functional titration in pure plasma. The relatively high Ca^{2+} concentration in the plasma saturated the fluorescence of Fluo-5N (working concentration: 1 μM, in 96-well plate), rendering it unsuitable for the titration system. **b,** Determination of K_d for Fluo-5N by Ca^{2+} titration. Hill fitting was used to determine the K_d value (90 μM). **c**, Competitive titration of Fluo-5N-Gd by LanND in the presence of Ca^{2+} interference. **d**, Competitive titration of Fluo-5N-Gd by LanND under varying pH conditions. **e**, Competitive titration of Fluo-5N-Gd by LanND with temperature changes. **f**, Competitive titration of Fluo-5N-Gd by LanND with interference by BSA or diluted plasma. All measurements were performed in biological triplicate and data are presented as mean ± SEM. Source data are provided as a Source Data file.

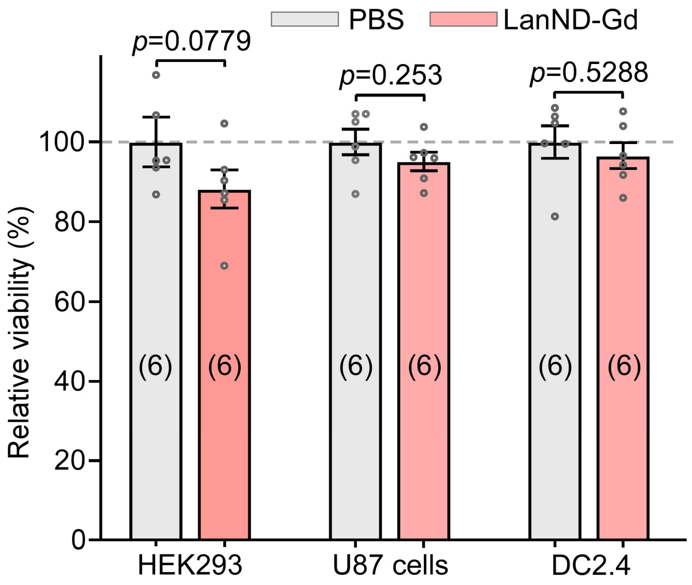

Supplementary Fig. 12 | Evaluation of cytotoxicity of LanND-Gd. The viability of HEK293, U87, and mouse DC2.4 cell lines after co-incubation with 100 μM PBS or LanND-Gd was analyzed by MTT assay. Data are expressed as mean ± SEM. No noticeable differences were observed in cell viability between the test and control groups, as calculated by unpaired Student's *t* test (two-tailed with criteria of significance: $*p<0.05$). Source data are provided as a Source Data file.

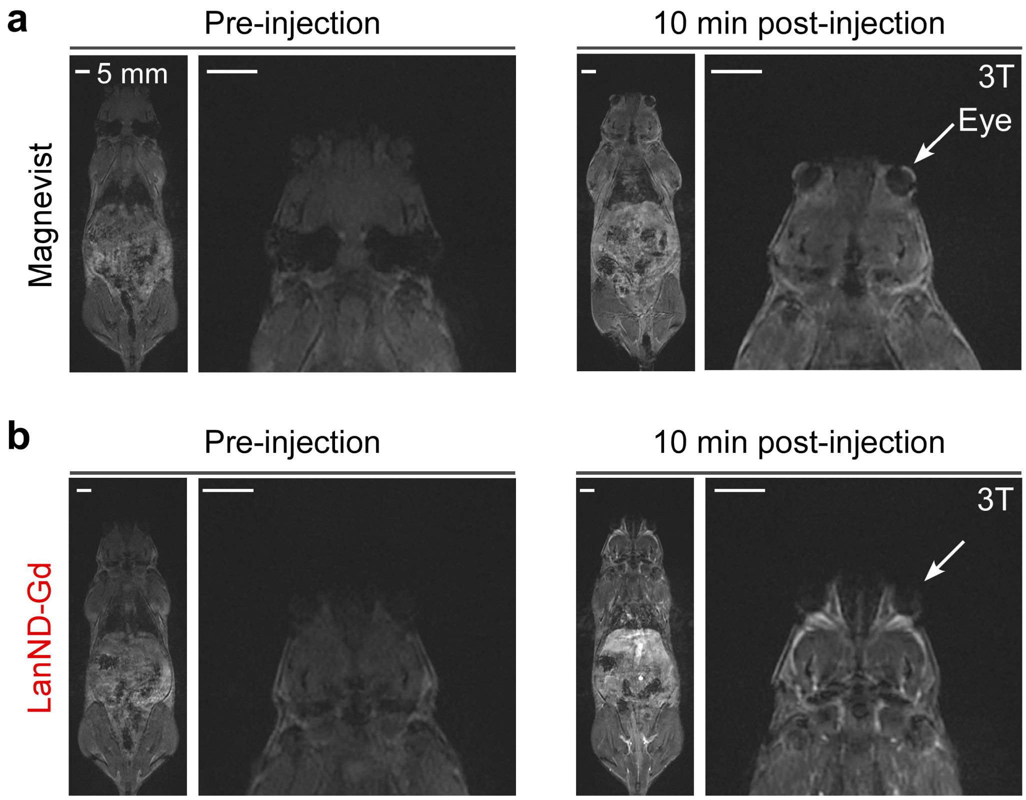

Supplementary Fig. 13 | Comparison of T_1-weighted eye images enhanced by Magnevist (a) or LanND-Gd (b) under a 3T scanner.

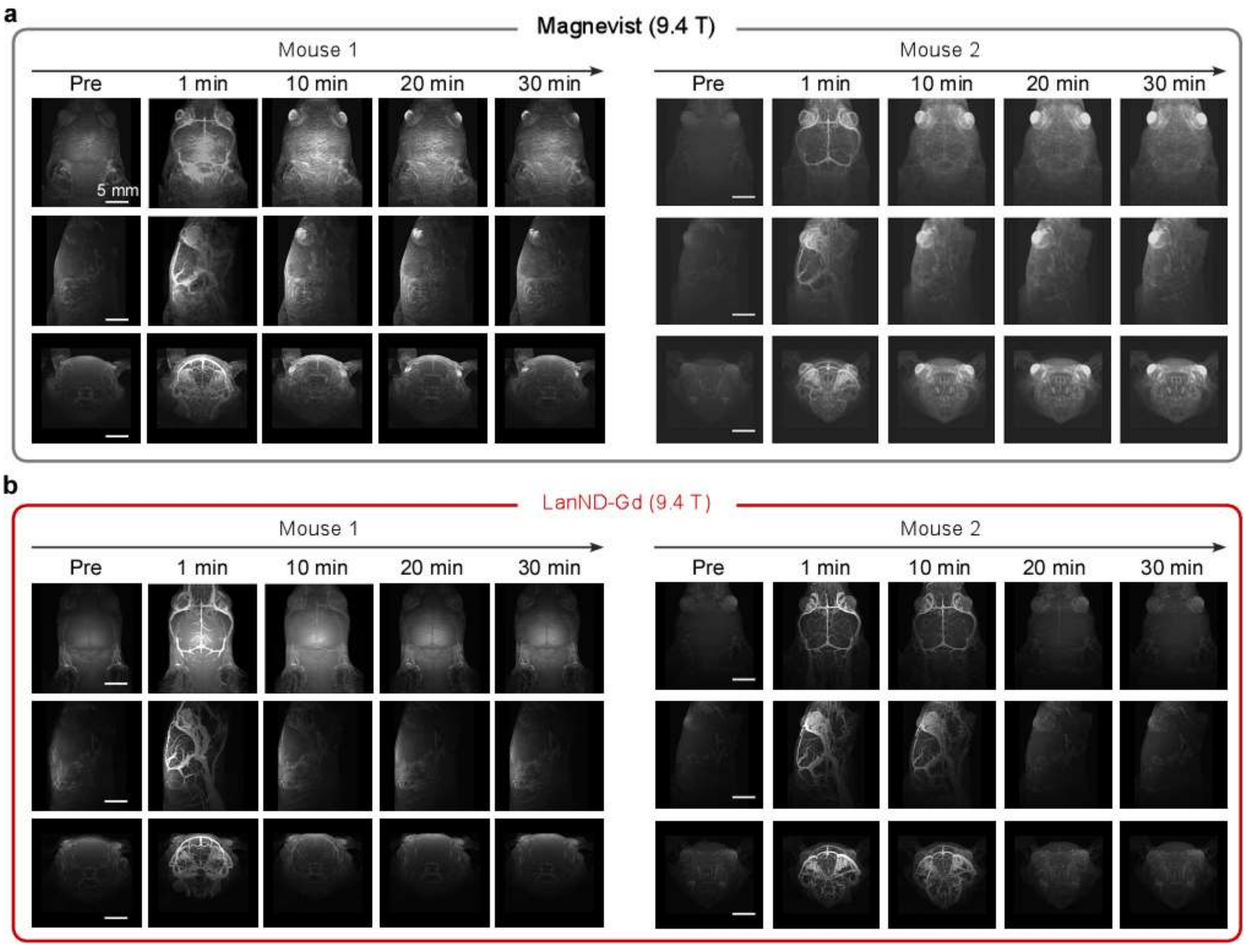

Supplementary Fig. 14 | Comparison of T_1-weighted brain images enhanced by Magnevist (a) or LanND-Gd (b) under a 9.4 T scanner. Images are displayed from different views, including longitudinal, side, and cross sections, at different time intervals following the agent injection.

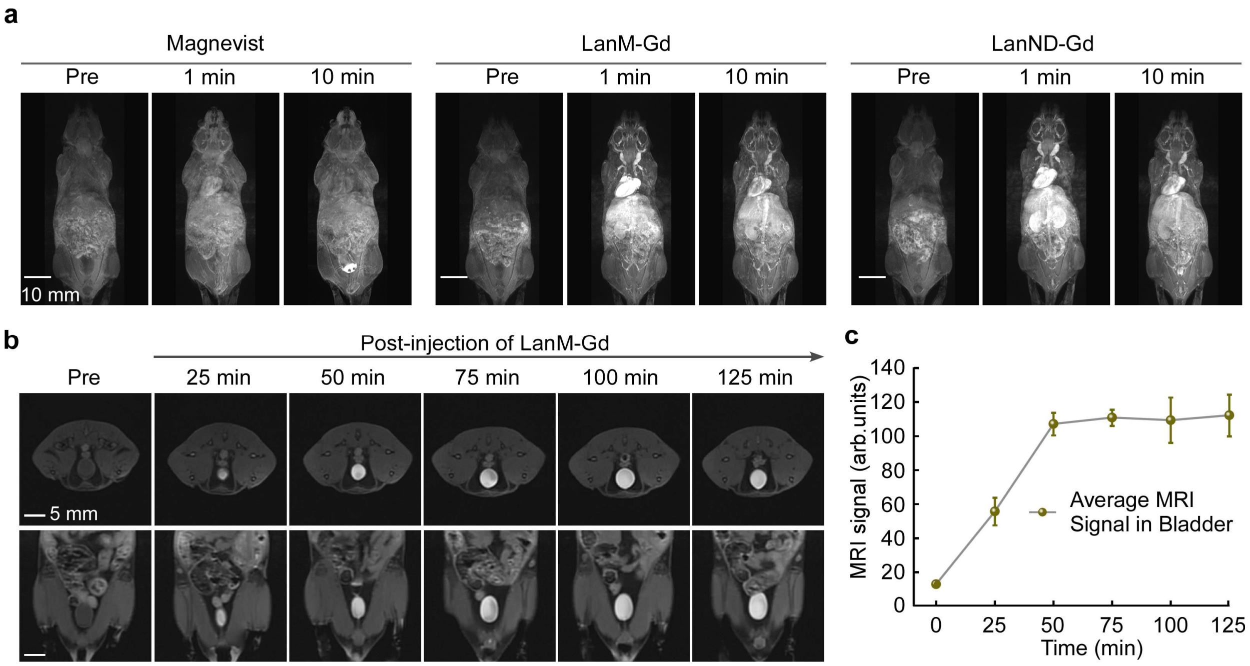

Supplementary Fig.15 | Whole-body imaging contrasted with different contrast agents. a, Representative images enhanced with Magnevist, LanM-Gd or LanND-Gd under a 3T scanner. **b,** Urinary excretion of LanM-Gd revealed by long-term bladder imaging. The top and bottom images display cross-sectional and vertical-sectional profiles, respectively. **c,** Statistical analysis of the average MRI signals in the bladder over time. Three mice were included, and data are presented as mean ± SEM. Source data are provided as a Source Data file.

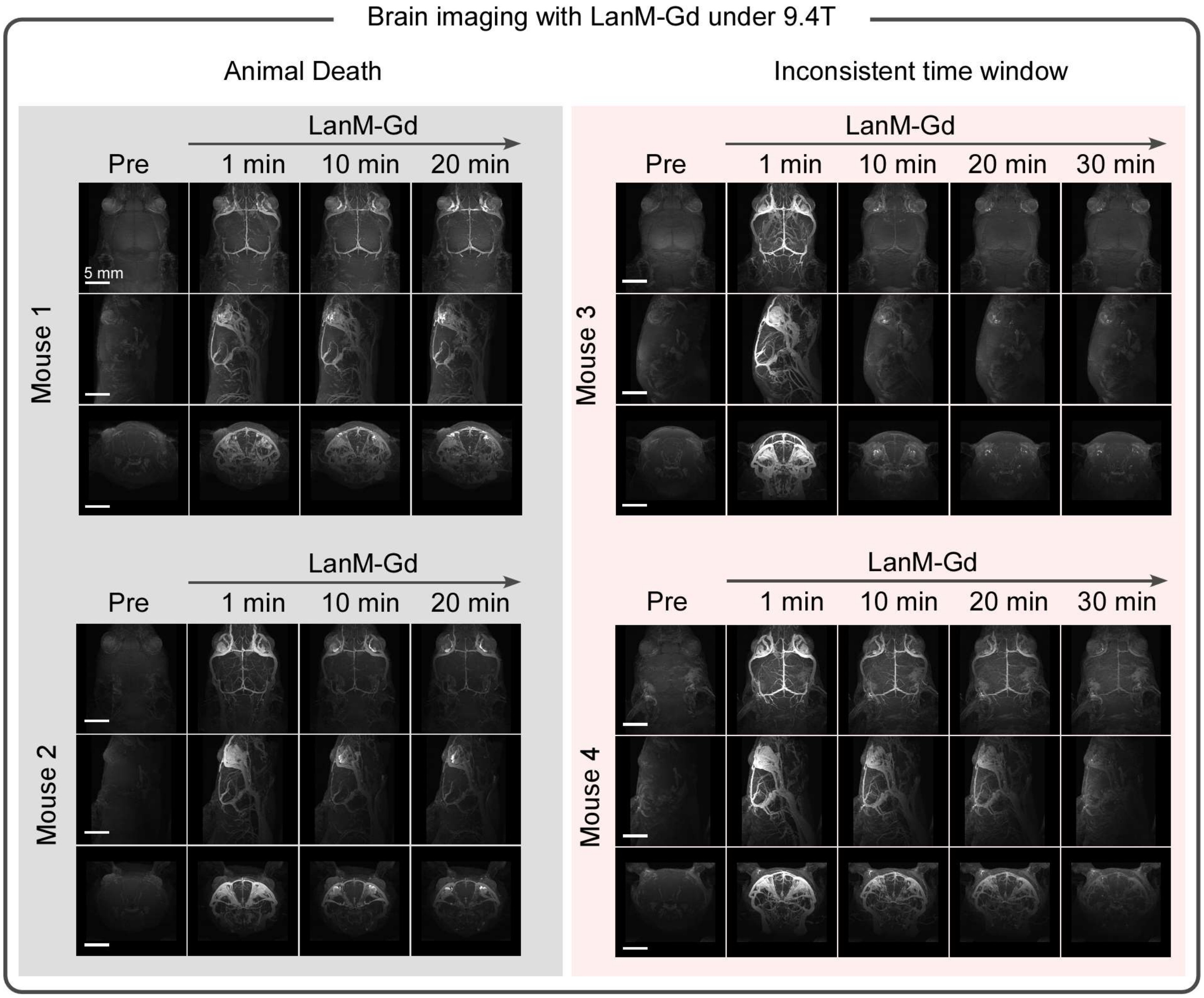

Supplementary Fig.16 | Brain imaging enhanced with LanM-Gd under 9.4T. Four mice were used for brain imaging, two of which died during the imaging process (left: mouse 1 and mouse 2), and the remaining two showed inconsistent results in terms of effective time windows (right: mouse 3 and mouse 4).

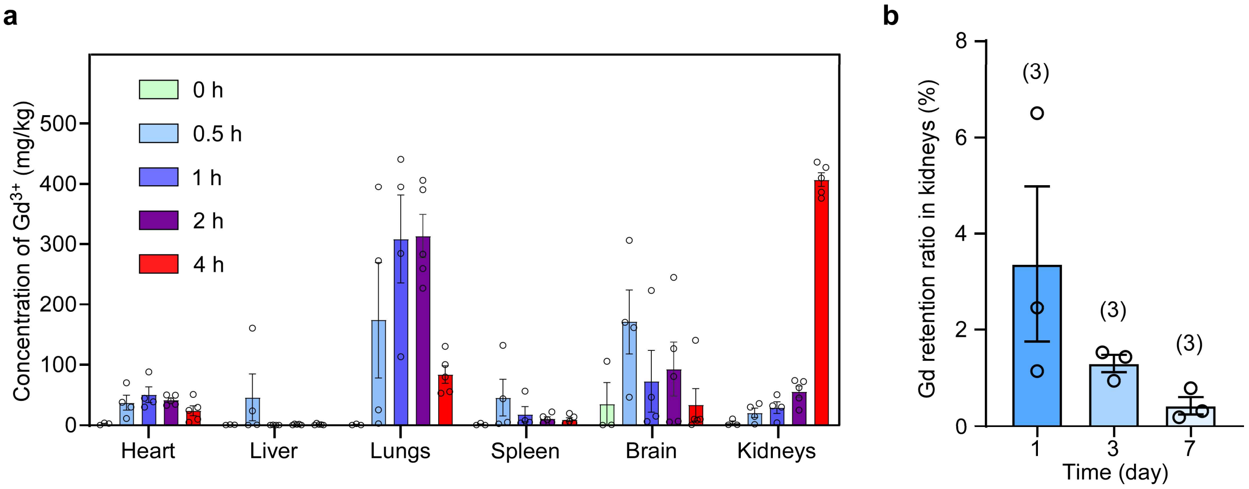

Supplementary Fig. 17 | Distribution of Gd^{3+} in various organs after injection of LanND-Gd. a, After LanND-Gd injection, Gd^{3+} concentrations in heart, liver, lungs, spleen, brain, and kidneys were measured by ICP-OES at different time points. Each group included at least three samples for statistical analysis: three mice for 0 h, four mice for 0.5 h and 1 h, five mice for 2 h and 4 h. **b,** Analysis of Gd retention ratio in kidneys on day1, day3 and day7. Data are presented as mean ± SEM and source data are provided as a Source Data file.

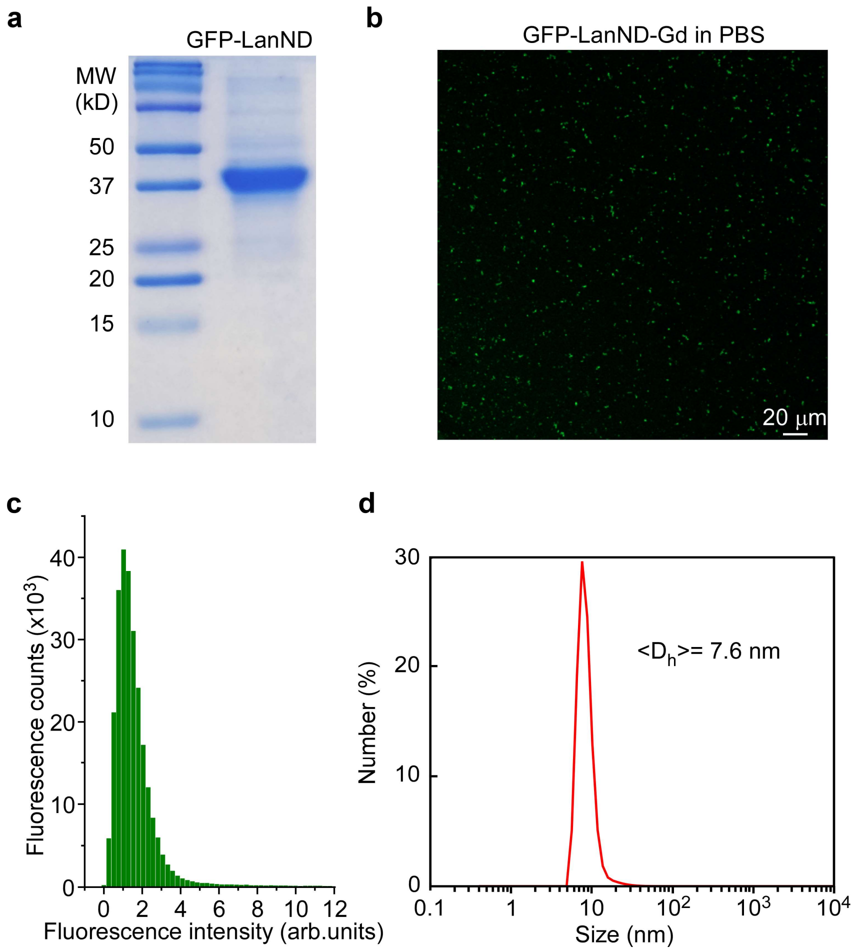

Supplementary Fig. 18 | Characterization of GFP-tagged LanND. a, SDS page-gel of purified GFP-LanND. Experiments were performed in biological triplicate, yielding similar results. **b,** Evaluation of GFP-LanND's dispersibility and luminescence using confocal microscopy. **c,** Statistical analysis of luminescent spots in b. **d,** Hydrodynamic size analysis for GFP-LanND-Gd using DLS. Experiments were performed in biological triplicate.

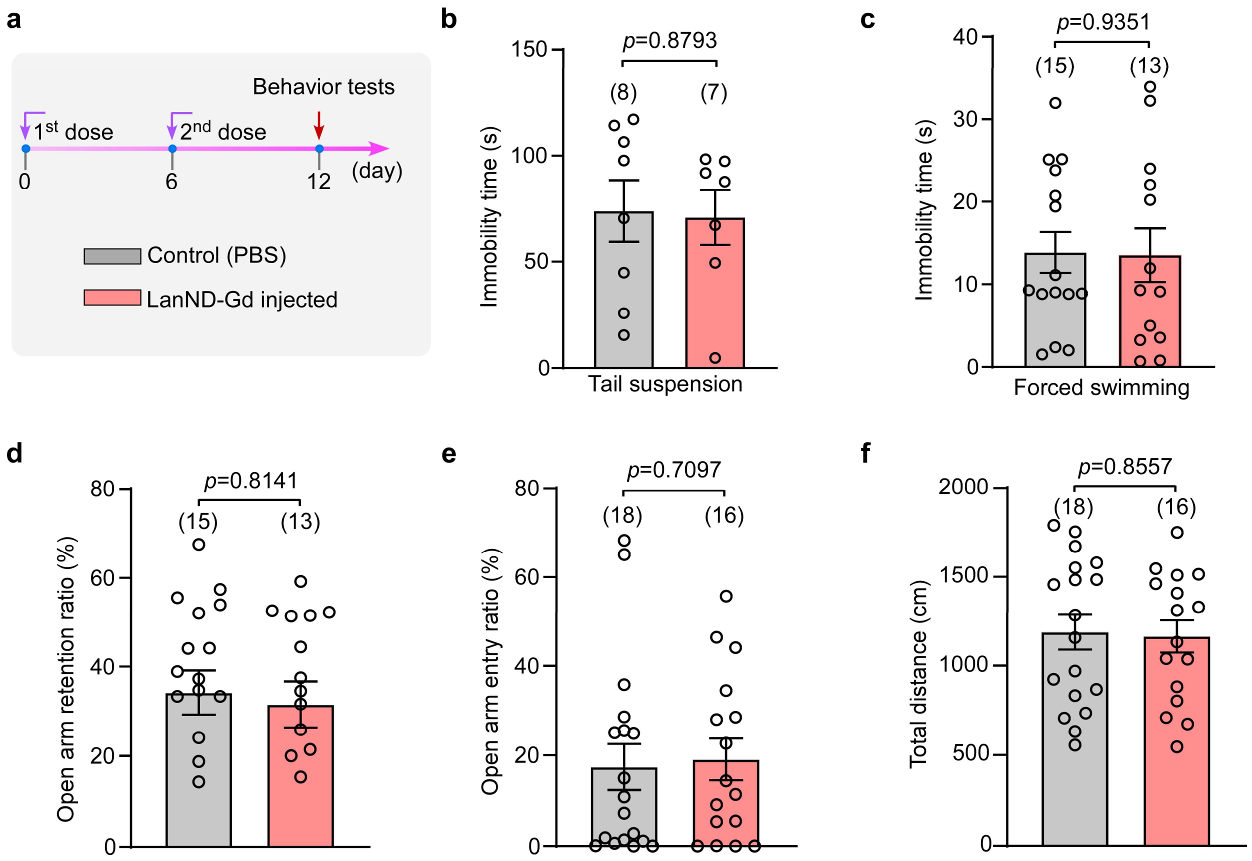

Supplementary Fig. 19 | Emotional test for potential immunogenic damage. a, Experimental timeline for behavior test. Protein agents were administered twice: the first dose on day 0 and a second dose on day 6, allowing a sufficient time to elicit immunogenicity. On day 12, depression and anxiety-related behavior tests were conducted. **b and c,** Evaluation of depression-like behavior using the tail suspension (b) and forced swim (c) test, respectively. **d-f,** Assessment of anxiety-like behavior using the elevated plus maze test. The parameters of open arm retention ratio (d), open arm entry ratio (e), and total distance (f) were statistically analyzed. Data are presented as mean ± SEM, and unpaired student's *t*-test (two-tailed with criteria of significance: $*p<0.05$) were calculated when applicable. Source data are provided as a Source Data file.

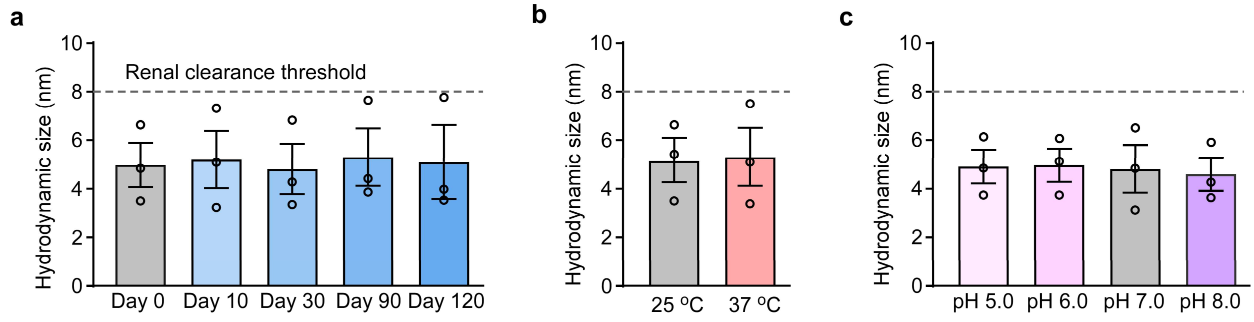

Supplementary Fig. 20 | Colloidal stability of protein-based agents under environmental variations. a-c, Hydrodynamic sizes of LanND-Gd measured under different time points (a), temperatures (b), and pH values (c), respectively. The size of the renal clearance threshold is indicated by the dashed line. Experiments were performed in biological triplicate and data are presented as mean ± SEM. Source data are provided as a Source Data file.

Uncropped gel for Supplementary Fig. S6b

Lane 6: protein ladder; Lane 7: LanM; Lane 8: LanM-Gd; Lane 9: LanND; Lane 10: LanND-Gd

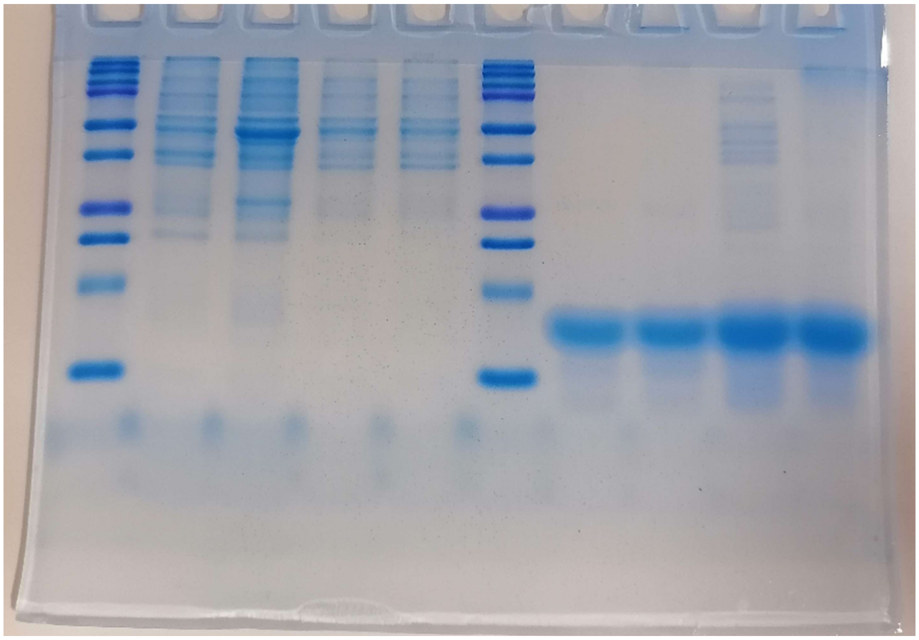

Uncropped gel for Supplementary Fig. S18a

Lane 8: protein ladder; Lane 9: GFP-LanND

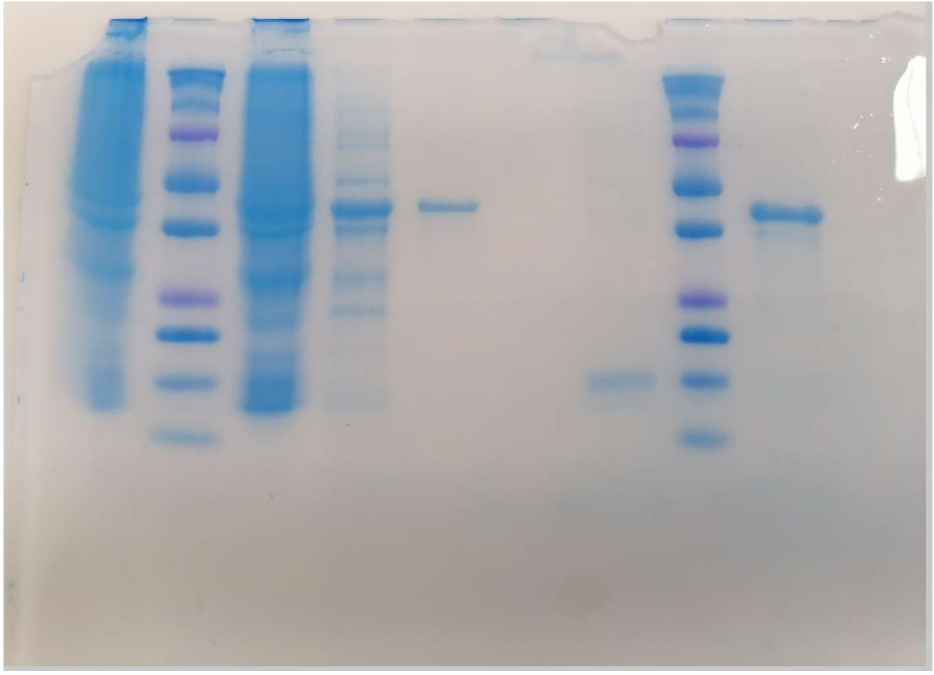

Supplement: Supplementary file 1 — Supplementary Information [file 41467_2024_54167_MOESM1_ESM.pdf]
