# Supplementary material for: Single-point mutated lanmodulin as a high-performance MRI contrast agent for vascular and kidney imaging
Source: Nat Commun. 2024 Nov 13;15:9834. doi: 10.1038/s41467-024-54167-4 (PMC11561317; doi:10.1038/s41467-024-54167-4)
Supplement: Supplementary file 2 — Description of Additional Supplementary Files [file 41467_2024_54167_MOESM2_ESM.pdf]

Description of Additional Supplementary Files

Supplementary Movie 1. 3D-reconstructed imaging before and after injecting LanND-Gd.

Supplementary Movie 2. 3D-reconstructed imaging for brain vasculatures.
