# Supplementary material for: Single-point mutated lanmodulin as a high-performance MRI contrast agent for vascular and kidney imaging
Source: Nat Commun. 2024 Nov 13;15:9834. doi: 10.1038/s41467-024-54167-4 (PMC11561317; doi:10.1038/s41467-024-54167-4)
Supplement: Supplementary file 5 — Reporting Summary [file 41467_2024_54167_MOESM5_ESM.pdf]

nature portfolio

Corresponding author(s): Xiaogang Liu

Last updated by author(s): Nov 2, 2024



# Reporting Summary

Nature Portfolio wishes to improve the reproducibility of the work that we publish. This form provides structure for consistency and transparency in reporting. For further information on Nature Portfolio policies, see our Editorial Policies and the Editorial Policy Checklist.

## Statistics

For all statistical analyses, confirm that the following items are present in the figure legend, table legend, main text, or Methods section.

| n/a | Confirmed | |
|---|---|---|
| ☐ | ☒ | The exact sample size ($n$) for each experimental group/condition, given as a discrete number and unit of measurement |
| ☐ | ☒ | A statement on whether measurements were taken from distinct samples or whether the same sample was measured repeatedly |
| ☐ | ☒ | The statistical test(s) used AND whether they are one- or two-sided<br>*Only common tests should be described solely by name; describe more complex techniques in the Methods section.* |
| ☐ | ☒ | A description of all covariates tested |
| ☒ | ☐ | A description of any assumptions or corrections, such as tests of normality and adjustment for multiple comparisons |
| ☒ | ☐ | A full description of the statistical parameters including central tendency (e.g. means) or other basic estimates (e.g. regression coefficient) AND variation (e.g. standard deviation) or associated estimates of uncertainty (e.g. confidence intervals) |
| ☐ | ☒ | For null hypothesis testing, the test statistic (e.g. $F$, $t$, $r$) with confidence intervals, effect sizes, degrees of freedom and $P$ value noted<br>*Give P values as exact values whenever suitable.* |
| ☒ | ☐ | For Bayesian analysis, information on the choice of priors and Markov chain Monte Carlo settings |
| ☒ | ☐ | For hierarchical and complex designs, identification of the appropriate level for tests and full reporting of outcomes |
| ☒ | ☐ | Estimates of effect sizes (e.g. Cohen's $d$, Pearson's $r$), indicating how they were calculated |

*Our web collection on statistics for biologists contains articles on many of the points above.*

## Software and code

Policy information about availability of computer code

| | |
|---|---|
| Data collection | MRI images were collected by uMR 790 or uMR 9.4T scanners from United Imaging Healthcare company, and images were analyzed by ImageJ. In vivo biodistribution imaging was performed using the IVIS system from Perkin Elmer. Neural bahavior data were analyzed by Matlab2021. Electrophysiological data were collected by pClamp11.2 software from Molecular Devices. |
| Data analysis | Data were analyzed with GraphPad Prism 10.1.2, Origin 2019, Clampfit (Axon pClamp software v.11.2), iQ3, ImageJ, PyMOL, Smart v3.0, Imaris 9.0.1 (Bitplane), and Matlab 2021. |

For manuscripts utilizing custom algorithms or software that are central to the research but not yet described in published literature, software must be made available to editors and reviewers. We strongly encourage code deposition in a community repository (e.g. GitHub). See the Nature Portfolio guidelines for submitting code & software for further information.

## Data

Policy information about availability of data

All manuscripts must include a data availability statement. This statement should provide the following information, where applicable:

- Accession codes, unique identifiers, or web links for publicly available datasets
- A description of any restrictions on data availability
- For clinical datasets or third party data, please ensure that the statement adheres to our policy

Protein sequence and structure data were retrieved from the Protein Data Bank: lanmodulin (6MI5 [https://doi.org/10.2210/pdb6MI5/pdb]). All the data used to generate figures in this paper are provided in the Supplementary Information and Source Data file. The full image dataset is available from the corresponding author

upon request. Source data are provided with this paper.

## Research involving human participants, their data, or biological material

Policy information about studies with human participants or human data. See also policy information about sex, gender (identity/presentation), and sexual orientation and race, ethnicity and racism.

| | |
|---|---|
| Reporting on sex and gender | *Use the terms sex (biological attribute) and gender (shaped by social and cultural circumstances) carefully in order to avoid confusing both terms. Indicate if findings apply to only one sex or gender; describe whether sex and gender were considered in study design; whether sex and/or gender was determined based on self-reporting or assigned and methods used. Provide in the source data disaggregated sex and gender data, where this information has been collected, and if consent has been obtained for sharing of individual-level data; provide overall numbers in this Reporting Summary. Please state if this information has not been collected. Report sex- and gender-based analyses where performed, justify reasons for lack of sex- and gender-based analysis.* |
| Reporting on race, ethnicity, or other socially relevant groupings | *Please specify the socially constructed or socially relevant categorization variable(s) used in your manuscript and explain why they were used. Please note that such variables should not be used as proxies for other socially constructed/relevant variables (for example, race or ethnicity should not be used as a proxy for socioeconomic status). Provide clear definitions of the relevant terms used, how they were provided (by the participants/respondents, the researchers, or third parties), and the method(s) used to classify people into the different categories (e.g. self-report, census or administrative data, social media data, etc.) Please provide details about how you controlled for confounding variables in your analyses.* |
| Population characteristics | *Describe the covariate-relevant population characteristics of the human research participants (e.g. age, genotypic information, past and current diagnosis and treatment categories). If you filled out the behavioural & social sciences study design questions and have nothing to add here, write "See above."* |
| Recruitment | *Describe how participants were recruited. Outline any potential self-selection bias or other biases that may be present and how these are likely to impact results.* |
| Ethics oversight | *Identify the organization(s) that approved the study protocol.* |

Note that full information on the approval of the study protocol must also be provided in the manuscript.

# Field-specific reporting

Please select the one below that is the best fit for your research. If you are not sure, read the appropriate sections before making your selection.

☒ Life sciences ☐ Behavioural & social sciences ☐ Ecological, evolutionary & environmental sciences

For a reference copy of the document with all sections, see nature.com/documents/nr-reporting-summary-flat.pdf

# Life sciences study design

All studies must disclose on these points even when the disclosure is negative.

| | |
|---|---|
| Sample size | No statistical predetermination of sample sizes were performed. Sample sizes used in this study were deemed to be representative and sufficient, which was based on the low variability of the results between biological replicates and on overall reproducibility. |
| Data exclusions | No data exclusions were conducted. |
| Replication | All experimental presented in this study were repeated at least three times, and most of them more than five times. All attempts at replication were successful. |
| Randomization | Randomization was performed for behavior, biodistribution and pharmacokinetics studies, where animals were randomly divided into different groups. |
| Blinding | Blinding was conducted for animal behavior experiments. Data collection and analysis were performed by different researchers. |

# Reporting for specific materials, systems and methods

We require information from authors about some types of materials, experimental systems and methods used in many studies. Here, indicate whether each material, system or method listed is relevant to your study. If you are not sure if a list item applies to your research, read the appropriate section before selecting a response.

## Materials & experimental systems

| n/a | Involved in the study | |
|---|---|---|
| ☒ | ☐ | Antibodies |
| ☐ | ☒ | Eukaryotic cell lines |
| ☒ | ☐ | Palaeontology and archaeology |
| ☐ | ☒ | Animals and other organisms |
| ☒ | ☐ | Clinical data |
| ☒ | ☐ | Dual use research of concern |
| ☒ | ☐ | Plants |

## Methods

| n/a | Involved in the study | |
|---|---|---|
| ☒ | ☐ | ChIP-seq |
| ☒ | ☐ | Flow cytometry |
| ☐ | ☒ | MRI-based neuroimaging |

# Eukaryotic cell lines

Policy information about cell lines and Sex and Gender in Research

| | |
|---|---|
| Cell line source(s) | HEK293 cell line, obtained from ATCC. |
| Authentication | The cell line was periodically authenticated by STR profiling. |
| Mycoplasma contamination | The cell line was periodically tested for mycoplasma contamination. |
| Commonly misidentified lines (See ICLAC register) | No commonly misidentified cell lines were used. |

# Animals and other research organisms

Policy information about studies involving animals; ARRIVE guidelines recommended for reporting animal research, and Sex and Gender in Research

| | |
|---|---|
| Laboratory animals | C57BL/6J mice aged 8-10 weeks were used. |
| Wild animals | The study did not involve wild animals. |
| Reporting on sex | Male. |
| Field-collected samples | The study did not involve any samples collected from field. |
| Ethics oversight | Animals for MR imaging and behavior test in this study were conducted with the approval of the Animal Care and Use Committee of Shenzhen Institute of Advanced Technology, Chinese Academy of Science (animal protocol number: SIAT-IACUC-220921-YGS-SZH-A2188).. The mice were purchased from SPF Biotechnology Co., Ltd (China).<br>Pharmacokinetics studies were approved by the National University of Singapore Institutional Animal Care and Use Committee (IACUC; animal protocol number: R22-0407) and conformed to the guidelines on the care and use of animals for scientific purposes (NACLAR, Singapore, 2004) and the Guide for the Care and Use of Laboratory Animals published by the US National Institutes of Health (NIIH Publication, 8th Edition, 2011). These mice were purchased from InVivos (Singapore).<br>Mice were kept in a controlled environment, with a consistent 12 /12-h light-dark cycle and unrestricted access to standard food and water in cages for 5-6 mice. Ambient temperature was maintained at 23-25 °C. |

Note that full information on the approval of the study protocol must also be provided in the manuscript.

# Plants

| | |
|---|---|
| Seed stocks | *Report on the source of all seed stocks or other plant material used. If applicable, state the seed stock centre and catalogue number. If plant specimens were collected from the field, describe the collection location, date and sampling procedures.* |
| Novel plant genotypes | *Describe the methods by which all novel plant genotypes were produced. This includes those generated by transgenic approaches, gene editing, chemical/radiation-based mutagenesis and hybridization. For transgenic lines, describe the transformation method, the number of independent lines analyzed and the generation upon which experiments were performed. For gene-edited lines, describe the editor used, the endogenous sequence targeted for editing, the targeting guide RNA sequence (if applicable) and how the editor was applied.* |
| Authentication | *Describe any authentication procedures for each seed stock used or novel genotype generated. Describe any experiments used to assess the effect of a mutation and, where applicable, how potential secondary effects (e.g. second site T-DNA insertions, mosiacism, off-target gene editing) were examined.* |

# Magnetic resonance imaging

## Experimental design

| | |
|---|---|
| Design type | Whole-body and brain imaging. |
| Design specifications | T1-weighted 3T or 9.4T imaging. |
| Behavioral performance measures | Biodistribution and metabolic route of the developed MRI contrast agent |

## Acquisition

| | |
|---|---|
| Imaging type(s) | Standard MRI. |
| Field strength | 3T and 9.4T |
| Sequence & imaging parameters | For animal T1-weighted imaging, the 3T preclinical MRI system with the following specifications: radiofrequency coil, special small animal coil (mouse coil); TR, 600 ms; TE, 12.16 ms; FOV, 60 mm × 30 mm; FA, 145°; voxel size, 0.47 mm × 0.23 mm × 0.6 mm; matrix size, 256 × 256; bandwidth/pixel, 260 Hz/pixel; slice thickness, 0.6 mm; number of averages, 8; number of slices, 10.<br>Parameters for 9.4T brain imaging: coil, dedicated mouse brain surface coil; scanning sequence, GR; TR=10.39 ms; TE=1.75 ms; FOV=18 × 22 mm2; NEX=1; FA=20°; Voxel size=0.1 × 0.1 × 0.1 mm3; Matrix size=224 × 183; slice thickness=0.1 mm; Number of slices=160; Bandwidth/pixel=195 Hz/pixel; oversampling=10%; Echo train length=1. |
| Area of acquisition | Kidney and brain |

Diffusion MRI ☐ Used ☒ Not used

## Preprocessing

| | |
|---|---|
| Preprocessing software | ImageJ |
| Normalization | Yes |
| Normalization template | Normalized by the region without contrast agent |
| Noise and artifact removal | No |
| Volume censoring | No |

## Statistical modeling & inference

| | |
|---|---|
| Model type and settings | Unpaired student' t test. |
| Effect(s) tested | Not applicable. |

Specify type of analysis: ☐ Whole brain ☐ ROI-based ☒ Both

Anatomical location(s): Brain, kidneys, bladder.

| | |
|---|---|
| Statistic type for inference (See Eklund et al. 2016) | Not applicable. |
| Correction | Not applicable. |

## Models & analysis

| n/a | Involved in the study |
|---|---|
| ☒ | ☐ Functional and/or effective connectivity |
| ☒ | ☐ Graph analysis |
| ☒ | ☐ Multivariate modeling or predictive analysis |
